# Upregulation of SLUG expression in canine mammary gland tumors and its prognostic significance

**DOI:** 10.1186/s12917-023-03646-9

**Published:** 2023-08-08

**Authors:** Soo-Bin Cheon, Wan Hee Kim

**Affiliations:** Department of Veterinary Clinical Sciences, College of Veterinary Medicine and Research Institute for Veterinary Science, Seoul National University, 1 Gwanak-Ro, Gwanak-Gu, Seoul, 08826 Republic of Korea

**Keywords:** Dogs, Mammary gland tumor, SLUG protein, Immunohistochemistry

## Abstract

**Background:**

SLUG (also known as snai2), which is a transcription factor in epithelial–mesenchymal transition (EMT), plays an important role in tumorigenesis. Several human studies have revealed that SLUG expression downregulates E-cadherin activity to induce metastasis and invasion of tumor cells, and its association with tumor mechanisms is under constant evaluation. In clinical veterinary medicine, one study revealed upregulated SLUG expression in canine oral squamous cell carcinoma. However, the association between canine mammary gland tumors (MGT), the most common neoplasm in intact female dogs, and SLUG has not been investigated yet. Therefore, this study aimed to evaluate the differences in SLUG expression among canine normal mammary gland tissue and MGTs using immunohistochemistry. In addition, its prognostic significance was evaluated by analyzing the correlation with the Ki-67 proliferation index and various clinicopathological features.

**Results:**

SLUG expression increased substantially from normal mammary gland tissues to MGTs, especially showing the strongest expression in malignant MGT than in benign MGT. Negative SLUG expression was observed in mostly normal mammary gland tissues, whereas all tissues in malignant MGT showed positive SLUG expression. Furthermore, positive SLUG expression was associated with higher Ki-67 index, larger tumor size (> 3 cm), and metastasis. Kaplan–Meier survival curve analysis revealed that positive SLUG expression was significantly associated with poor overall and disease-free survival.

**Conclusions:**

These results indicate that SLUG is upregulated in canine MGTs and positive SLUG expression is positively correlated with poor prognosis. Thus, SLUG protein can be a novel biomarker and therapeutic target for canine patients with MGT.

## Background

Canine mammary gland tumors (MGT) are the most common neoplasms in sexually intact, female dogs and account for 50–70% of all tumors in female dogs [1]. Dogs with benign MGT have an increased risk of developing subsequent malignant tumors [1, 2]. The incidence of MGT varies among regions; however, three main factors in increasing MGT risks have been identified through various studies and these include age, hormonal exposure and breed [1]. In addition, the histologic type of the mammary tumor influences the choice of treatment. For example, surgery is the main treatment choice for MGT, whereas adjuvant therapy should be considered first for inflammatory mammary carcinoma [3]. Thus, the ability to stage and define mammary tumors before initiating therapy is strongly recommended.

Hormonal exposure plays an important role in the development of MGTs. Many of these MGTs are associated with hormone receptors (HR), including the estrogen receptor (ER) and progesterone receptor (PR) [4, 5]. Although the results vary in studies of canine MGT, tumor expression of ER and PR is associated with a more positive outcome [6–8]. In human breast cancer, endocrine therapy is strongly recommended for patients with ER-positive tumors [9]. In addition, endocrine therapy in humans has been considered because of the association of epithelial–mesenchymal transition (EMT) with endocrine resistance in breast cancers. Indeed, transcription factors involved in EMT, such as the SNAI family, have recently been demonstrated to play a pivotal role in tumorigenesis.

When EMT is activated, the downregulation of epithelial-specific proteins (such as E-cadherin and occludin) leads to the acquisition of mesenchymal properties. The loss of epithelial cell–cell adhesion induces increased cell migration, and thus, tumor invasiveness and metastasis [10]. The induction of mesenchymal properties facilitates the upregulation of vimentin and fibronectin, cytoskeletal reorganization, and resistance to cell apoptosis. These features are associated with cancer progression and aggressiveness [11].

In studies on human breast cancer, expression of SLUG, an SNAI family member also known as SNAI2, increased in breast cancers compared to normal mammary epithelium; and SLUG expression had a strong correlation with the loss of E-cadherin during EMT [11, 12]. In addition, increase in SLUG expression in human breast cancer was correlated with higher metastasis and a higher tumor grade [13].

Moreover, high SLUG expression is associated with poor clinical outcomes in human gastric cancer [14], esophageal squamous cell carcinoma [15, 16], colorectal carcinoma [17], lung adenocarcinoma [18], and prostate cancer [19]. To the best of our knowledge, SLUG has been studied only in association with canine oral squamous cell carcinoma [20] and melanoma in a mouse model [21]. Furthermore, human breast cancers are correlated with high SLUG expression. Therefore, in this study, SLUG expression in canine mammary glands was investigated, and its expression in normal canine mammary glands, benign tumors, and malignant tumors was compared using immunohistochemistry (IHC). Moreover, we evaluated the correlation between SLUG and the clinicopathological features of dogs to find a potential prognostic factor.

## Results

### Clinical information

A total of 84 dogs were included in the study. Table 1 shows that 21 dogs had normal mammary gland tissue and were intact females, with the majority being Beagles (*n* = 19); 29 dogs had benign MGTs, of which three were spayed females; and 34 dogs were diagnosed with malignant MGTs, of which nine were spayed females. The histopathological types of MGTs were categorized according to Goldschmidt et al. [22]. Benign and malignant MGTs were categorized as simple, complex, or mixed. Adenosquamous tumors were also categorized separately as it is a special type of epithelial malignant MGTs. No sarcomas, carcinosarcoma or other special types of malignant neoplasm was included in this study. Histological grades were categorized according to Pena et al. [23]. Among the 34 dogs with malignant MGTs, seventeen were of histological grade 1, nine of grade 2, and eight of grade 3. The major breeds of MGTs patients were Maltese (*n* = 18) and Yorkshire terriers (*n* = 11).

**Table 1:** Clinical parameters of the dogs included in this study

	**Normal mammary glands** **(*n* = 21)**	**Benign tumors** **(*n* = 29)**	**Malignant tumors** **(*n* = 34)**
**Median age (years; range)**	2 (2)	11 (5–15)	13 (6–16)
**Sex (n)**			
**Spayed female**	0	3	9
**Female**	21	26	25
**Breed (n)**	Beagle (21)	Yorkshire Terrier (7)	Yorkshire terrier (4)
		Maltese (11)	Maltese (7)
		Shih tzu (2)	Shih tzu (4)
		Schnauzer (1)	Schnauzer (1)
		Chihuahua (1)	Jindo (1)
		Cocker spaniel (1)	Cocker spaniel (5)
		Beagle (1)	Dachshund (2)
		Samoyed (1)	Marinoise (1)
		Poodle (1)	Poodle (7)
		Mixed (3)	Mixed (2)
**Histologic type (n)**	**-**	Adenoma, simple (10)	Carcinoma, simple (18)
		Adenoma, complex (14)	Carcinoma, complex (10)
		Mixed (5)	Carcinoma, mixed (5)
			Adenosquamous carcinoma (1)
**Histologic grade (n)**	**-**	-	Grade 1 (17)
	**-**	-	Grade 2 (9)
	**-**	-	Grade 3 (8)

### SLUG expression in mammary gland tissues and tumors

**Fig. 1:**
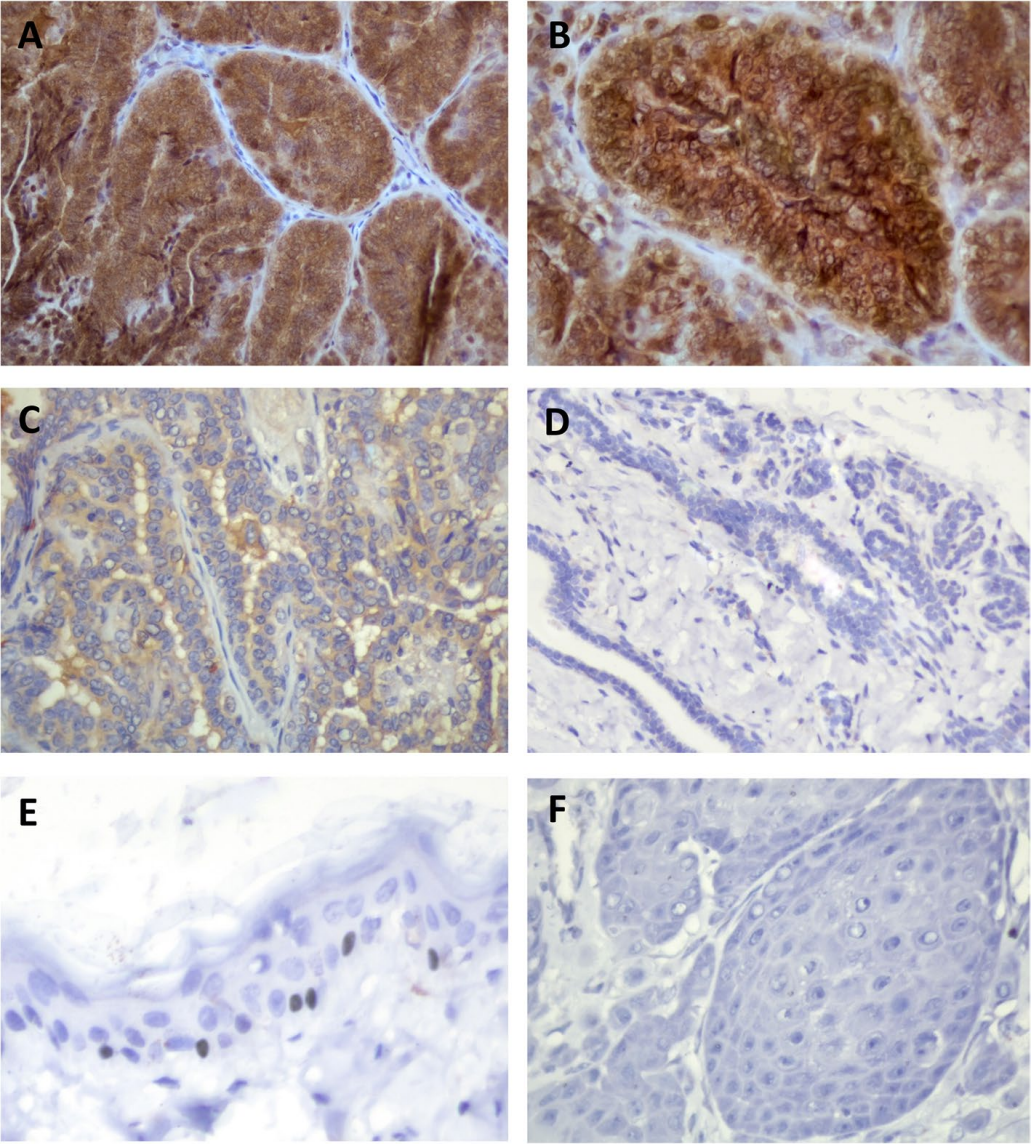
Immunohistochemical staining of SLUG in canine mammary gland tumors (MGT) tissues. (A, B) Malignant MGT showing strong SLUG staining. (C) Benign MGT showing moderate SLUG staining. (D) Normal mammary gland tissue showing negative SLUG staining. (E) In the positive control, SLUG expression is observed in the basal keratinocytes of the mouse skin. (F) In the negative control, no specific staining is observed. Original magnification: A, C, D, E, F: 400 × , B:1000 ×

Immunohistochemical staining of SLUG showed that it was mainly expressed in the cytoplasm of epithelial cells in the mammary glands. Specific immunostaining of SLUG was confirmed in the basal keratinocytes of mouse skin as a positive control **(**Fig. 1E**)**. The negative control did not exhibit SLUG expression **(**Fig. 1F**).** Positive SLUG expression was observed in all (100%) malignant MGT, in 97% of benign MGT and in 86% of normal mammary gland tissues. MGTs had higher positive SLUG expression compared with normal mammary gland tissues (*P* = 0.020). Furthermore, the staining intensity of SLUG increased from normal mammary gland tissues to benign and malignant MGT. (*P* = 0.022) (Table 2**, **Fig. 1A-D). Of the malignant MGTs, 65% showed high-SLUG expression.

**Table 2:** SLUG expression in normal, benign, and malignant mammary gland tumors

SLUG expression n (%)	Normal (*n* = 21)	Benign (*n* = 29)	Malignant (*n* = 34)	*P*-value
Negative	3 (14%)	1 (3%)	0 (%)	0.020 * for negative vs. positive
Positive				
Low (0–3)	13 (62%)	11 (38%)	12 (35%)	0.022 *For low vs. high
High (4–9)	5 (24%)	17 (59%)	22 (65%)	

*P < 0.05, indicates a statistically significant linear association between tumor malignancy and SLUG expression

### Correlation of clinicopathological factors and SLUG expression

Table 3 shows the correlations between various clinicopathological factors and SLUG expression. Among the tumors that showed positive SLUG expression in benign and malignant mammary tumors, those larger than 3 cm had higher SLUG expression than those smaller than 3 cm (*P* = 0.032). Consistently, 70% of malignant MGT was > 3 cm in a study by Sorenmo et al., indicating the increased risk in malignancy as the tumor diameter increases. Therefore, SLUG expression is associated with larger tumor size, a feature with recognized prognostic value according to Sorenmo et al. [2].

**Table 3:** Clinicopathological variables associated with SLUG expression

	**Number of tumors**	**SLUG expression**			
		**Low**	**High**	**Absent**	***p*-value**
**Tumor size (n)**					
≤ 3 cm	34	15	19		0.032 *
> 3 cm	28	8	20		
**Benign tumor (n)**					
Simple	10	4	6	0	0.522
Complex	14	5	9	0	
Mixed	5	2	2	1	
**Malignant tumor (n)**					
Simple	18	6	12		1.000
Complex	10	4	6		
Mixed	5	2	3		
Adenosquamous	1	0	1		
**Histological grade (n)**					
Grade 1	17	7	10		0.334
Grade 2	9	4	5		
Grade 3	8	1	7		
**Ki-67 (n)**					
≤ 15%	8	7	1		0.001 *
> 15%	26	5	21		
**Metastasis (n)**					
No	19	10	9		0.030 *
Yes	15	2	13		

*P < 0.05, indicates a statistically significant association between positive SLUG expression and clinicopathological variables

In addition, among the malignant MGTs, the correlation between SLUG expression, Ki-67 level (*P* = 0.001), and presence of metastasis during the follow-up period (*P* = 0.030) was statistically significant. Of the 26 malignant MGTs that showed a Ki-67 index of > 15%, the majority (80%, 21/26 tumors) presented high SLUG expression. SLUG expression showed no statistically significant correlation within each separate tumor groups of histological types and with histological grades within malignant tumors. In malignant tumors, 89% of simple carcinoma, 60% of complex carcinoma, and 60% of mixed carcinoma showed high-receptor expression. Furthermore, the one sample with histological diagnosis of adenosquamous carcinoma showed high-SLUG expression.

### Correlation of clinical outcome and SLUG expression

Disease-free survival (DFS) and overall survival (OS) of the 34 malignant MGTs were analyzed using Kaplan–Meier analysis. A total of 25 dogs died, of which 16 died due to causes related to MGTs.

Malignant tumors were analyzed using Kaplan–Meier analysis to address correlations between known prognostic factors, including tumor grade, stage, and metastasis, with survival time. As a result, statistically significant association was identified between overall survival with tumor grade, metastatic presence, and stage (*P* = 0.006, *P* < 0.001, *P* = 0.014, respectively). The results are consistent with studies that analyzed known prognostic factors with malignant tumors.

**Fig. 2:**
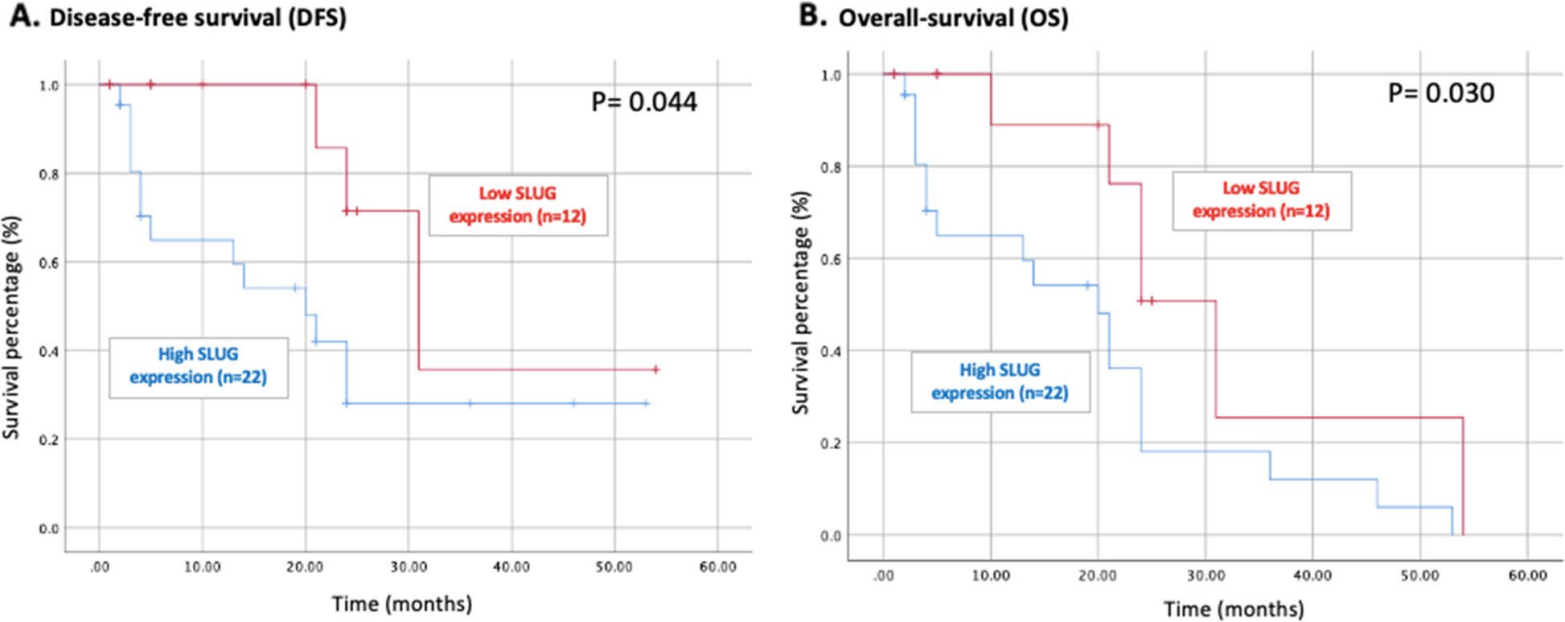
Kaplan–Meier survival curve of (A) disease-free survival and (B) overall-survival based on the SLUG expression of 34 dogs with malignant mammary gland tumors (median disease-free survival, 24 months; median overall survival, 21 months)

Malignant MGTs were classified into two groups of SLUG expression: high (*n* = 22) and low expression (*n* = 12). DFS and OS showed a statistically significant association between the two groups (*P* = 0.044 and *P* = 0.030, respectively). Patients with high SLUG expression had substantially slower DFS and poorer prognosis. The 2-year survival rate was 18% and 50.8% in the high and low SLUG expression group, respectively. The median DFS and OS was 24 and 21 months, respectively. (Fig. 2).

## Discussion

EMT is a normal developmental process during morphogenesis and plays a critical role in tumor invasion and metastasis [10]. Markers, such as TWIST, ZEB, SNAIL, and SLUG, are capable of initiating EMT. During this transition, the downregulation of E-cadherin leads to a loss of cell–cell adhesion, thereby increasing cell migration capacity and invasiveness, which is frequently observed in carcinomas [11]. Karen et al. [12] revealed a strong correlation between SLUG expression and loss of E-cadherin in human breast cancers. SLUG (SNAI2), one of the key EMT markers that is a member of the SNAIL zinc-finger family, is more highly expressed in breast cancer cells than in normal mammary cells [13]. Moreover, many studies have focused on and identified the regulation of EMT by SLUG during breast tumorigenesis; suggesting an association between high SLUG expression and a poorer prognosis. To our knowledge, this is the first veterinary study to evaluate the association between SLUG and canine mammary gland tissue and cancer cells.

Immunostaining of SLUG indicated that it was mainly expressed in the cytoplasm of mammary epithelial tumor cells and was partially expressed in the nuclei of some tumor cells. This pattern is similar to that observed in Gu et al., where IHC indicated a higher SLUG expression in human breast carcinoma than in normal mammary gland tissue. In terms of oral squamous cell carcinoma (SCC), this pattern is slightly different from that in canine oral SCC studied by Noguchi et al., where SLUG expression is mainly observed in the nucleus and partly in the cytoplasm [20]. However, in human studies of oral SCC, SLUG expression was detected in 71% of tumor cell cytoplasm [15]. The immunostaining disparity of SLUG expression in oral SCC between canine and human needs further research. In addition, specific immunostaining of SLUG was confirmed in the basal keratinocytes of mouse skin as a positive control (Fig. 1E) [24–26]. The disparity of SLUG immunostaining among mouse basal keratinocytes (nuclear), canine mammary gland tumors (mainly cytoplasmic in this study), and canine oral SCC (mainly nuclear) requires further clarification. Previous literatures also show different SLUG immunostaining in canine mammary glands and human breast cancers. According to Raposo-Ferreira et al., positive SLUG in canine mammary carcinomas was considered as those with nuclear immunostaining, although the cytoplasm was also stained [24]. This is in accordance with the study by Sarah Phillips et al., where the author mentions that the SNAG domain is essential for protein’s nuclear localization [11]. However in other studies of human breast cancer, SLUG expression has been shown in the cytoplasm mainly [25] and, in another study, in membranous and cytoplasmic immunostaining [26]. In epidermal keratinocytes, SLUG is known to bind to enhancer box elements in the nuclear E-cadherin promoter to repress transcription [27]. In epithelial cells, especially in tumor cells, some SLUG factors bind to the transmembrane proteins such as the tight junctions and the occludins leading to loss of cell–cell adhesions [28]. In addition, some studies have speculated that SLUG may also regulate subcellular localization of E-cadherin by redistributing them from adherens junctions into cytoplasmic compartments [27, 29]. Overall, the differences in SLUG immunostaining among different organs of the canine needs further research.

Our data indicated an increased expression of SLUG in canine MGT compared to normal mammary gland tissues (*P* = 0.020), consistent with previous human studies [13]. In the current study, differences in low and high SLUG expression groups was correlated to an increase in malignancy from normal tissues to malignant tumors (*P* = 0.022). SLUG functions in regulating cell differentiation, cell-state dynamics, and mammary stem cell state [11]. These SLUG-dependent processes are believed to play pivotal roles in breast tumor initiation and progression.

The correlation between SLUG expression and several clinicopathological factors, known to be prognostic markers in canine MGT, was assessed. Tumor size was correlated with SLUG expression in both benign and malignant tumors. According to MacEwen et al., tumor diameters larger than 3.4 cm have substantially worse outcomes for the dog than smaller tumors. In the current study, a statistically significant correlation between SLUG overexpression, metastatic status, and Ki-67 index was found. This is consistent with a human study where high SLUG expression in primary human breast tumors was correlated with increased metastatic potential [13, 26]. The correlation between SLUG overexpression and metastasis can be explained by the impact of the downregulation of E-cadherin, to which SLUG binds. This results in repression of integrin expression, leading to reduced cell adhesion. Subsequently, the capacity for cell migration increases and metastasis is initiated in local and distant regions.

In the current study, no significant correlation was detected between SLUG expression and histological grade, which is inconsistent with the findings of Liu et al. [26]. This discrepancy may be due to the small sample size and the fact that most patients with malignant tumors had low-grade tumors. In future studies, a larger sample size should be assessed to validate the impact of SLUG expression on tumor grade, similar to several human studies that have validated this correlation [15, 30]. Lastly, unlike the study from Siwen Gu et al., no correlation between the histopathological type of the tumor and SLUG expression in canine MGTs was identified in this study. This disparity may be due to the fact that the study from Siwen Gu et al. classified breast cancers only into “invasive ductal carcinoma (IDC)” and “others”. Moreover, several studies have found that in human and mouse mammary epithelium, SLUG is mainly localized to the basal/ myoepithelial cell layer than in the luminal layer [11]. This suggests that SLUG may regulate the epithelial-cell state and thus may be related to histologic types of tumor based on the origin. However, further investigation in the correlation of SLUG expression and histological type of MGT is needed in the future.

Our findings demonstrate that elevated SLUG expression is associated with poorer DFS and OS. Gu et al. [25] analyzed human breast cancer and found that the OS of patients with low SLUG expression was better than that of patients with high SLUG expression. However, no correlation was found for DFS in that study. In our analysis of the Kaplan–Meier survival curve, the 2-year survival rate between dogs with low and high SLUG expression was different (18.0% vs. 50.8%). Thus, our study suggests the potential of SLUG as a prognostic marker in dogs with MGTs. Additionally, human studies have confirmed a poor prognosis with elevated SLUG expression not only in breast cancer, but also in esophageal squamous cell carcinoma [15], colorectal carcinoma [17] and lung adenocarcinoma [18].

With cancer therapy and its relationship to EMT, numerous human studies are underway to evaluate the association of SLUG with cancer treatment. For example, patients with prostate cancer with low E-cadherin and high SLUG expression were found to have poorer outcomes, such that targeted therapy against specific regulators may be particularly beneficial [19]. In addition, Alves et al. reported that the knockdown of SLUG induced mesenchymal–epithelial transition (MET), reducing cell motility [30]. This offers insights into the role of SLUG as a potential target for therapeutic strategies against EMT in human breast cancer. Moreover, SLUG is a regulator of the mammary stem cell state in both breast tumor and normal cells. Bhat-Nakshatri et al. found that breast cancer cells that overexpressed SLUG had higher proportions of CD44^+^/CD24^−^ cancer stem cells, indicating that SLUG-induced transcriptional programs lead to stemness [31]. Hence, similar to the studies in humans, SLUG may become the next targeted therapy for aggressive MGTs in dogs. The relationship between canine MGTs and high SLUG expression investigated in the present study is the first step in validating the mechanisms and therapies associated with SLUG.

This study has several limitations. First, the sample size for each group was small. In analyzing the correlation between histological grade and SLUG expression, most of the dogs had low-grade tumors, which could have been the reason for the lack of correlation between the two factors. Future studies need to include larger sample sizes to validate and allow for an accurate, multi-perspective analysis. Second, all the dogs in normal mammary gland tissue group were 2 years old, which is relatively younger than the patients’ age with mammary gland tumor. However, it was difficult to obtain normal mammary gland tissues from old dogs with no other diseases. In future studies, age ranges should be similar among all groups. Third, since not all the samples that died during the study proceeded to necropsy, due to the owner’s refusal, it is not possible to confirm with absolute certainty the existence/absence of metastatic diease. Lastly, this study did not evaluate the SLUG mRNA and protein levels. Additional methods, such as western blotting and real-time PCR, are required to fully comprehend and verify the relationship between SLUG expression and canine MGTs. However, this study will serve as a starting point for future analyses of SLUG and canine MGTs.

## Conclusions

This study indicated a relationship between SLUG expression and canine MGTs. Compared to normal mammary gland tissues, mammary gland tumors showed higher SLUG expression; along with malignant MGT expressing the highest SLUG expression. In addition, statistical significance was confirmed between SLUG overexpression and several prognostic factors, such as tumor size, metastasis, and Ki-67 index. Finally, our study established a correlation between high SLUG expression and poor DFS and OS in canine MGT. These results indicate that SLUG may be a prognostic marker for canine MGT. However, further investigations are required to comprehensively understand the mechanisms of SLUG and other EMT regulators involved in the invasion and metastasis of canine MGT.

## Methods

### Tissue samples

The majority of normal mammary gland tissue samples were obtained from 21, 2-year-old intact female Beagles housed in the Department of Veterinary Surgery, College of Veterinary Medicine at Seoul National University (SNU). Mammary gland tissue samples were collected after euthanasia (using 10% potassium chloride under anesthesia with 0.6 ml/kg propofol) unrelated to cancer in another experiment (SNU-200709–4-5). Physical examination, blood analysis (serum chemistry, complete blood count), radiography, and abdominal ultrasonography were performed in the Veterinary Medical Teaching Hospital (VMTH) of SNU to confirm that all Beagles were healthy. In addition, none of the dogs in the normal mammary gland tissue group were pregnant, and a vaginal smear was used to confirm that the dogs were in the anestrus period. All the samples were immediately fixed in 10% neutral formalin at room temperature (20–25 °C) for 48 h. Subsequently, they were embedded in paraffin blocks and sectioned at 4-µm thickness using a microtome (Leica Microtome HM355S, Plymouth, MN, USA). Finally, the sections were stained with hematoxylin and eosin (H&E) to histologically confirm normal mammary gland tissue.

MGT samples (29 benign and 34 malignant) were collected from the dogs that underwent surgery for resection of MGT between January 2018 and December 2021 at SNU-VMTH. A portion of the tumor was collected and immediately fixed in 10% neutral formalin at room temperature (20–25 °C) for 48 h. Similar to the normal tissue samples, these were embedded in paraffin blocks and sectioned at 4-µm thickness using a microtome (Leica Microtome HM355S, Plymouth, MN, USA), followed by H&E staining. The remainder of the tumor was diagnosed as MGT based on histopathological examination at the veterinary pathology laboratory of SNU or IDEXX laboratory. In addition, histological type and grade of the mammary gland tumors were analyzed by the according veterinary pathology laboratory of SNU or IDEXX laboratory.

All owners were informed and approved of the study procedure and purpose. Clinical data on these animals, tumor size, metastasis, and latest status at follow-up were also collected for prognostic analysis. Malignant tumors were graded according to the criteria described by Goldschmidt et al. [32], and, similar to the study by Sorenmo et al. [1], if a dog was identified with more than one MGT, the tumor with the most aggressive pathological features was chosen. The pathologic features was ordered by the following criteria: malignant was chosen over benign tumors primarily, and in cases with several malignant tumors, the tumor with highest histologic grade was selected. Patients with other types of cancer or those who had undergone adjuvant therapy, such as chemotherapy or radiotherapy, were excluded from the study. The protocol, procedures, and the reporting of animal experiments employed were ethically reviewed and approved according to ARRIVE guidelines and Institutional Animal Care and Used Committee (IACUC) of Seoul National University (SNU-220323–3).

### Immunohistochemistry (IHC)

Mammary tissues were fixed in 10% neutral formalin and embedded in paraffin blocks. For IHC, paraffin-embedded tissues were sectioned at 4-µm thickness using a microtome (Leica Microtome HM355S). After fully drying the sections at room temperature (20–25 °C), they were deparaffinized with xylene (cat. 115, Duksan, Gyeong-gi, South Korea) twice for 5 min each, then rehydrated in graded alcohol for 3–5 min sequentially (100% twice, 90%, 80%, and 70%). Antigen retrieval was performed using 10 mM citrate acid buffer (pH 6) in a 2100-retriever pressure cooker (PickCell Laboratories, Amsterdam, the Netherlands) for 20 min. Endogenous peroxidase activity was quenched by incubating with 0.3% H_2_O_2_ for 30 min at room temperature (20–25 °C). The sections were then treated with normal goat serum (diluted to 2.5%; ab7481; Abcam, Cambridge, MA, USA) for 20 min to block non-specific binding. The sections were then incubated overnight at 4 °C with mouse anti-SLUG monoclonal antibody (1:100; sc-166476, Santa Cruz Biotechnology, CA, USA) and rabbit anti-Ki-67 polyclonal antibody (1:500; PA5-19,462, Invitrogen). For the negative control, sections were incubated with mouse IgG isotype control (1:100; 31,903, Invitrogen, Paisley, UK) for 30 min. The antibody against SLUG has been validated in canine tissues [20]. Thereafter, the sections were incubated with secondary antibodies for 1 h: goat anti-mouse biotinylated IgG (H + L) (ab64255, Abcam, Cambridge, Massachusetts, USA) for samples with primary anti-SLUG antibody and negative control samples, and horseradish peroxidase (HRP) goat anti-rabbit IgG (H + L) (1:1000; 32,460, Invitrogen, Paisley, UK) for ki-67 samples. The slides were then treated with 3,3'-diaminobenzidine tetrahydrochloride diaminobenzidine substrate chromogen system (DAKO) for 1 min. The slides were then washed with distilled water and counterstained with Mayer's hematoxylin. Normal C57BL/6 J mouse skin tissue was used as a positive control [27, 33, 34]. Immuno-stained slides were scanned using an Olympus BX51 microscope (Olympus, Tokyo, Japan) with the appropriate light filters (Tucsen, Fuzhou, China).

### Quantification of IHC staining

IHC was quantified and evaluated by two observers using a weighted histoscore. SLUG is mainly stained in the cytoplasm; thus, cytoplasmic staining was considered a positive result. Five representative fields of vision at 400 × magnification were selected to evaluate SLUG expression.

The grading of positive cells was performed according to a previous study on SLUG expression in breast cancer [25].The proportion scores were determined as 0 (0–4%), 1 (5–24%), 2 (25–50%), and 3 (> 50%). Staining intensity was scored as 0 (negative), 1 (mild), 2 (moderate), and 3 (strong). The final histoscore was calculated by multiplying the proportion and intensity scores. The average value of the five high-power fields in each tissue was used as the final score. The median of all histoscores was 4; thus, samples with a score of $\ge 4$ were considered to have high expression, and samples with a score < 4 were considered to have a low expression.

Ki-67 nuclear staining was performed. All positively stained cells in 10 representative high-power fields containing 1000 cells were counted. A high Ki-67 index value was defined as ≥ 15% nuclear staining, according to the method of Kadthur et al. [35], in which canine MGTs were divided into low- and high-risk groups to analyze MGT prognosis.

### Follow-up data

All dogs were evaluated before surgery, 2-weeks post-surgery, and every 3–6 months at VMTH-SNU. Metastasis and tumor recurrence were evaluated using physical examination, thoracic radiography (three views), abdominal ultrasound, and fine-needle aspiration. Computed tomography, biopsy, and autopsy were performed if required. When new abdominal tumors were detected, or lymph nodes were enlarged, fine-needle aspiration or excisional biopsy was performed to rule out other neoplasms; otherwise, it was considered to be local recurrence or metastatic disease.

### Statistical analysis

All statistical analyses were performed using the SPSS software version 25.0 (IBM Corp., Armonk, NY, USA). The correlation of SLUG expression and various clinicopathological parameters was measured using Fisher's exact, Mann–Whitney U, chi-square, and linear-by-linear association tests. Low and high SLUG expression in malignant MGTs were compared because all malignant samples showed positive expression.

Kaplan–Meier survival curves were plotted and compared using the log-rank test. DFS was defined as the time from the date of primary surgical treatment to the time of detection of the first local recurrence or metastasis. Samples lost to follow-up, those with no recurrence or metastasis until the end of the study, or those that died were censored. OS was defined as the interval from the date of primary surgical treatment to death associated with MGTs. Samples lost to follow-up due to death that was unrelated to MGTs or those alive until the end of the study were all censored. Statistical significance was set at *P* < 0.05.

## Authors’' contributions

Conceptualization: SBC and WHK; data curation: SBC and WHK; formal analysis: SBC and WHK; funding acquisition: WHK; investigation: SBC and WHK; methodology: SBC and WHK; supervision: WHK; visualization: SBC; writing – original draft: SBC; and writing – review and editing: SBC and WHK. All authors have read and approved the final manuscript.

## Data Availability

The datasets used and/or analyzed during the current study are available from the corresponding author upon reasonable request.
